# ADM-SLAM: Accurate and Fast Dynamic Visual SLAM with Adaptive Feature Point Extraction, Deeplabv3pro, and Multi-View Geometry

**DOI:** 10.3390/s24113578

**Published:** 2024-06-02

**Authors:** Xiaotao Huang, Xingbin Chen, Ning Zhang, Hongjie He, Sang Feng

**Affiliations:** 1 School of Electromechanical Engineering, Guangdong University of Technology, Guangzhou 510006, China; gdutfsae_hxt@126.com (X.H.);; 2 Guangdong Productivity Promotion Center, Guangzhou 510075, China

**Keywords:** V-SLAM, dynamic environments, feature point extraction, semantic segmentation, multi-view geometry

## Abstract

Visual Simultaneous Localization and Mapping (V-SLAM) plays a crucial role in the development of intelligent robotics and autonomous navigation systems. However, it still faces significant challenges in handling highly dynamic environments. The prevalent method currently used for dynamic object recognition in the environment is deep learning. However, models such as Yolov5 and Mask R-CNN require significant computational resources, which limits their potential in real-time applications due to hardware and time constraints. To overcome this limitation, this paper proposes ADM-SLAM, a visual SLAM system designed for dynamic environments that builds upon the ORB-SLAM2. This system integrates efficient adaptive feature point homogenization extraction, lightweight deep learning semantic segmentation based on an improved DeepLabv3, and multi-view geometric segmentation. It optimizes keyframe extraction, segments potential dynamic objects using contextual information with the semantic segmentation network, and detects the motion states of dynamic objects using multi-view geometric methods, thereby eliminating dynamic interference points. The results indicate that ADM-SLAM outperforms ORB-SLAM2 in dynamic environments, especially in high-dynamic scenes, where it achieves up to a 97% reduction in Absolute Trajectory Error (ATE). In various highly dynamic test sequences, ADM-SLAM outperforms DS-SLAM and DynaSLAM in terms of real-time performance and accuracy, proving its excellent adaptability.

## 1. Introduction

In the research field of intelligent robotics and autonomous navigation systems, Simultaneous Localization and Mapping (SLAM) technology plays a crucial role [1,2]. Notably, the work of M.W. M. Gamini Dissanayake and others in 1990, based on the Extended Kalman Filter (EKF) [3], laid a solid theoretical foundation for subsequent SLAM research.

Over the past three decades, SLAM technology has evolved from basic theoretical research to a key technology in various application scenarios and has transformed from a simple localization and mapping technology into a complex system integrating various sensors and algorithms. Among these sensors, visual sensors have received special attention in the field of Visual SLAM (V-SLAM) due to their low cost, high resolution, and rich information acquisition capabilities. The ORB-SLAM [4] uses sparse features, demonstrating the enormous potential of visual SLAM in practical applications. Subsequently, methodologies such as PTAM [5], LSD-SLAM [6], DSO [7], ORB-SLAM2 [8], VINS Mono [9], DM-VIO [10], and ORB-SLAM3 [11] emerged, significantly advancing the field of visual SLAM.

However, extracting key information from visual data in complex and dynamic real-world environments is challenging. Dynamic objects, such as pedestrians and moving vehicles, add complexity to SLAM systems. These dynamic objects can mislead the SLAM system, resulting in incorrect map or trajectory estimations. Therefore, accurate detection and segmentation of dynamic objects have become critical tasks in V-SLAM.

In recent years, the development of deep learning techniques has opened up new possibilities for handling dynamic scenarios. For instance, DynaSLAM [12] is a visual SLAM system that utilizes ORB-SLAM2 as its foundation. It detects dynamic objects by combining multi-view geometry and deep learning techniques. Additionally, it fills in the scene occluded by dynamic objects by utilizing background restoration techniques. On the other hand, DS-SLAM [13] combines a semantic segmentation network and a movement consistency checking method. DS-SLAM not only reduces the influence of dynamic objects, thus greatly improving the localization accuracy in dynamic environments, but also generates a dense semantic octree map that can be used for advanced task processing. Guangqiang Li et al. [14] have significantly advanced deep learning in the SLAM domain through the implementation of multi-task networks. Simultaneously, Xiqi Wang et al. [15] have notably enhanced RGB-D SLAM accuracy in dynamic environments. Furthermore, innovations like RDS-SLAM [16] and AHY-SLAM [17] have made substantial contributions to evolving deep learning techniques within SLAM.

However, these algorithms have the disadvantage of high computational complexity, hardware requirements, and slow processing speed when applying large deep learning models, particularly in real-time application scenarios. To enhance the real-time performance, robustness, and accuracy of SLAM systems in dynamic environments, this paper proposes ADM-SLAM, based on ORB-SLAM2. The ADM-SLAM comprises three modules: an adaptive feature point homogenization extraction module, a lightweight deep learning semantic segmentation module, and a multi-view geometric segmentation module.

When existing SLAM algorithms perform dynamic environment perception, they are limited by the uneven distribution of feature points in visual image data. These dynamic objects will be regarded as part of the static environment, resulting in errors in map construction and positioning. At the same time, using deep learning semantic segmentation models for dynamic scene segmentation requires a lot of computing power, and the processing speed is slow and inefficient. In addition, due to the changeable motion status of dynamic objects in SLAM application scenarios, the feature discrimination method of dynamic object feature areas is not clear enough. To address such problems, the SLAM algorithm needs to be able to adaptively and uniformly extract feature points, and at the same time, have lightweight and efficient dynamic scene segmentation capabilities and effectively remove non-feature dynamic feature points. The ADM-SLAM algorithm proposed in this study not only improves the real-time performance and accuracy in dynamic environments but also maintains higher operating efficiency and a clearer way of identifying dynamic object features. It is expected to solve the limitations of existing SLAM technology in dynamic environments, promote technological progress in related fields, and provide more powerful and reliable environment perception capabilities for future intelligent systems.

The work in this paper has the following main contributions:

(1) Enhanced adaptive feature point homogenization: We introduce a novel application of the adaptive non-maximal suppression algorithm, SSC (Suppression via Square Covering), which ensures uniform redistribution of feature points across the image, addressing the issue of non-uniform local distribution.
(2) Optimized lightweight semantic segmentation: We enhance the Deeplabv3pro deep learning semantic segmentation network to be more lightweight and efficient, allowing for faster and more accurate segmentation of dynamic objects without the heavy computational load.
(3) Advanced multi-view geometric segmentation: We implement a multi-view geometric approach to accurately determine the motion state of objects and perform secondary detection of dynamic targets, effectively removing dynamic feature points from the feature region.

In Section 2, we review the development of feature point extraction algorithms and the evolution of visual SLAM in dynamic scenes. Section 3 details the architecture of the ADM-SLAM algorithm. Section 4 presents an evaluation of the system’s real-time per-formance, robustness, and accuracy using the TUM RGB-D dataset [18]. Finally, Section 5 concludes the paper with a summary and discussion of the findings.

## 2. Related Work

### 2.1. Homogenized Feature Point Extraction Algorithm

In SLAM, feature point extraction is one of the core techniques to provide robots with critical information about the environment. SIFT [19] is highly acclaimed for its scale invariance and robustness to rotational and illumination changes, setting a benchmark. However, due to its computational intensity, the Speeded-Up Robust Feature (SURF) has been proposed and applied [20]. After that, ORB (Oriented FAST and Rotated BRIEF) was proposed [21], which is more computationally efficient than its predecessor.

Although ORB is a popular choice, particularly in real-time SLAM systems like ORB-SLAM2, it presents some challenges. The most significant of these is the uneven distribution and clustering of ORB feature points in the image. This inhomogeneity results in suboptimal image matching accuracy and wide variations in camera pose estimation. To address this issue, researchers have been working on improving ORB detection algorithms. Mur-Artal et al. [22] utilized a quadtree segmentation algorithm to divide the image into multiple regions, resulting in a more even distribution of feature points. Yu et al. [23] took it a step further by enhancing the traditional quadtree algorithm with region edge detection and feature point redundancy to further improve uniformity.

However, solely improving algorithms may not be enough to tackle the inherent challenges of ORB. The development of Adaptive Non-Maximum Suppression (ANMS) methods was facilitated by this realization. ANMS is a technique that ensures a uniform spatial distribution of key points, which is crucial in avoiding problems such as degraded configurations of motion or structure, or redundant information about clusters of points in SLAM. Brown et al. [24] were pioneers in this field, introducing ANMS to improve the robustness of image matching, particularly in applications such as panoramic stitching and other similar applications. 

However, the original ANMS had high computational complexity and was not suitable for real-time applications. Subsequent research has attempted to optimize ANMS. Cheng et al. [25] proposed an algorithm that uses a two-dimensional k-d tree for spatial segmentation, separates keypoints into rectangular image regions, and then selects the strongest features from each cell. Additionally, Gauglitz et al. [26] proposed two complementary methods with running times less than quadratic. The article describes two methods for feature point extraction in SLAM. The first method employs an approximate nearest neighbor algorithm, while the second method, called ‘Suppression by Disk Coverage (SDC)’, aims to enhance the performance of ANMS by simulating an approximate radius nearest neighbor query. The article highlights the ongoing efforts to improve the uniformity and robustness of feature point extraction in SLAM through the introduction and optimization of techniques such as ANMS.

### 2.2. Visual SLAM Based on Dynamic Scene Segmentation

The emergence of deep learning techniques has made dynamic scene segmentation a crucial development in improving visual SLAM. Meanwhile, CNNs play an important role in recognition identification [27,28]. Traditional SLAM systems frequently encounter difficulties in dynamic environments where moving entities result in imprecise mapping and localization. Dyna-SLAM [12] and its successor, Dyna-SLAM II [29], ingeniously integrate multi-view geometry and instance segmentation. The first part of the text addresses the limitations of individual approaches, specifically the challenges of detecting distant objects using multi-view geometry. The second part introduces a new feature matching technique for dynamic objects that optimizes the camera, feature points, and dynamic objects simultaneously. This dual approach improves the accuracy of camera pose estimation, which is crucial for dynamic vision SLAM.

DS-SLAM [13] enhances this field by enabling real-time operation on embedded systems for the first time. It combines semantic segmentation of keyframes with depth graph clustering, reducing overall runtime and demonstrating excellent performance in dynamic scene understanding.

Kim’s SimVODIS++ [30] architecture integrates visual odometry, object segmentation, and instance segmentation. By processing three consecutive frames during dynamic motion, this method can evaluate pose, depth, and instance segmentation masks simultaneously. It integrates a Convolutional Neural Network (CNN) into one framework, reducing computation time. Additionally, its self-attention module in data preprocessing enhances semantic segmentation capabilities.

Dym-SLAM [31] introduces a 4D system that uniquely utilizes time as a factor in evaluating dynamic objects. Instead of treating moving objects as outliers, Dym-SLAM constructs dense maps of static environments while capturing the motion of these dynamic entities.

RDS-SLAM [16] builds on ORB-SLAM3 [11] and incorporates parallel semantic segmentation without compromising processing speed. The model achieves real-time motion frequencies, demonstrating its potential for t-world applications.

However, despite recent advancements, challenges still persist in the field. For instance, while deep learning-based methods like RDS-SLAM can achieve real-time frequencies of up to 30 Hz. Moreover, there exists a trade-off between segmentation accuracy and computational time: deep learning has high accuracy when processing complex and dynamic objects but is limited by computing resources and is difficult to process in real time. In contrast, geometric methods are very efficient in terms of processing speed, but cannot achieve the effect of accurately segmenting dynamic environments. The combination of semantic and geometric methods presents a promising approach, and researchers are currently concentrating on practical applications that leverage their combined strengths.

## 3. Materials and Methods

**Figure 1:**
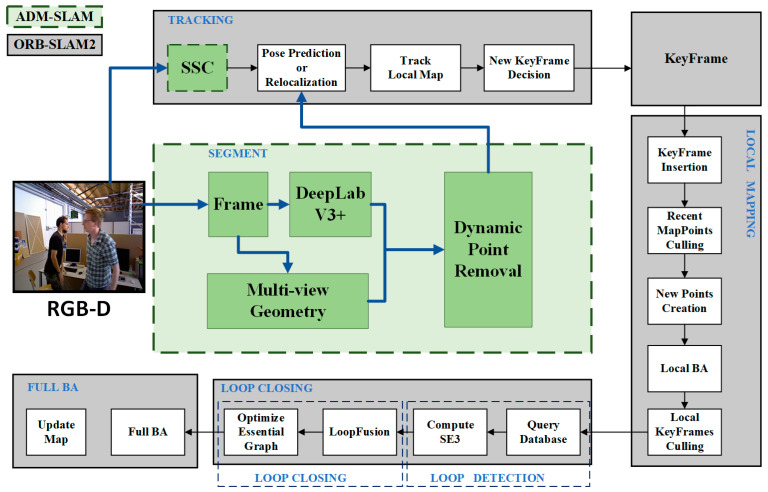
The schematic of the overall framework of the ADM-SLAM system.

ORB-SLAM2 is a versatile and precise Simultaneous Localization and Mapping (SLAM) algorithm that includes tracking, local mapping, backend optimization, and loop detection. Building on the foundation of ORB-SLAM2, this article proposes a dynamic environment SLAM system named ADM-SLAM. ADM-SLAM integrates adaptive feature point homogenization extraction (SSC) [32], semantic segmentation using Deeplabv3pro, and multi-view geometry [33]. Compared to ORB-SLAM2, the main improvement of ADM-SLAM is in its tracking module, which is better adapted to dynamic environments. This system provides more accurate environmental mapping and positioning, especially in rapidly changing scenes. The overall structure is illustrated in Figure 1.

The ADA-SLAM algorithm uses the RGB-D image data of the depth camera as input data. After algorithm processing, it generates the map environment data commonly used by the SLAM algorithm and its own running trajectory data. During the running process of the ADA-SLAM algorithm, by reading and processing RGB-D image data, and after processing by the feature extraction algorithm SSC and Deeplabv3pro, it generates its own positioning information and attitude prediction information. After key frame extraction, the tracking, local mapping, backend optimization, and loop detection processes are used to generate map environment data.

### 3.1. Adaptive Feature Point Homogenisation Extraction Module

The adaptive feature point homogenization extraction module introduces an algorithm called SSC (Suppression via Square Covering) to improve the local uniformity of feature points in the ORB-SLAM2 system. The SSC algorithm determines the optimal grid radius by dividing the image grid based on the number of target feature points and using a dichotomous method. This ensures that all feature points are arranged in order within the grid. Only the feature points within the specified radius of the grid are retained. The redundant feature points will be removed from the grid or the surrounding grids to achieve uniform distribution of feature points on the grid.

The specific implementation process of the SSC algorithm is:

1. Build an image pyramid to extract key point features at different scales.
2. In order to find the maximum radius $a_{h}$ and minimum radius $a_{l}$ of the square grid uniformly distributed on the image according to the image dimensions width $W_{1}$ and height $H_{1}$ (take the first frame of image as an example):
  (1) $$W_{1}=2a_{h}+\left(a_{h}+1\right)q-1$$
  (2) $$H_{1}=2a_{h}+\left(a_{h}+1\right)\left(l-1\right)$$

If q is the number of centroids in each row of squares, then there is q−1 distance between centroids. Similarly, l  is the number of centroids in each column of squares, so the centroids are l−1 away from each other.

Therefore, the number of centroids in each row and column are, respectively:

(3) $$q=\frac{W_{1}-a_{h}+1}{a_{h}+1}$$

(4) $$l=\frac{H_{1}-a_{h}+1}{a_{h}+1}$$

if the number of target extracted feature points is m, which is known, and l=m∕q, substituting Formulas (3) and (4) into it, the following equation is obtained:

(5) $$\left(m-1\right)a_{h}^{2}+a_{h}\left(W_{1}+2m-H_{1}\right)+m+W_{1}-H_{1}W_{1}=0$$

Solving the above equation, it will yield two solutions, one of which is always negative, and the other is the final estimated radius $a_{h}$ of the square is:

(6) $$a_{h}=-\frac{H_{1}+w_{1}+2m-\sqrt{\Delta }}{2\left(m-1\right)}$$

(7) $$\Delta =4W_{1}+4m+4H_{1}m+H_{1}^{2}+W_{1}^{2}-2W_{1}H_{1}+4H_{1}W_{1}m$$

When the feature points are located in a single square on the image and there is no space between the squares, the number of feature points at this time is recorded as n, and the number of feature points required for binary search is *m*. Since it is necessary to use a square with a side length of 2$a_{l}$, filling this image with squares to retrieve *m* feature points in these squares, the minimum radius $a_{l}$ for binary search should be:

(8) $$a_{l}=\frac{1}{2}\sqrt{\frac{n}{m}}$$

3. After obtaining the maximum radius $a_{h}$ and the minimum radius $a_{l}$, find the most suitable side length of the mesh betwee $a_{h}$ and $a_{l}$ by bisecting w:
  (9) $$w=2a_{l}+\left(2a_{h}-2a_{l}\right)∕2$$
4. After obtaining a grid uniformly distributed over the image, each feature point $k_{i}$ to be homogenized is traversed, the corresponding grid is selected for that feature point according to the resolution *c*, and all grid cells within the radius of the current feature point are marked as covered to prevent subsequent feature points from selecting these grid cells again, which helps to ensure a uniform distribution between key points and avoid selecting the same grid cells multiple times. These covered grid cells will be skipped in subsequent traversals, thus ensuring that the coverage of keypoints is even.
  (10) $$c=\frac{w}{2}$$
  (11) $$row_{i}=\frac{k_{iy}}{c}$$
  (12) $$col_{i}=\frac{k_{ix}}{c}$$
  c is the resolution of grid initialisation, $row_{i}$ is the horizontal coordinate of the grid where the feature point is located, $col_{i}$ is the vertical coordinate of the grid where the feature point is located.
5. The number of feature points derived from statistical screening determine whether the number of feature points meets the target number of feature points, and if not, skip to the third step until the target number of feature points is met.

### 3.2. Deeplabv3pro Semantic Segmentation Model

#### 3.2.1. Overall Architecture of Deeplabv3pro

**Figure 2:**
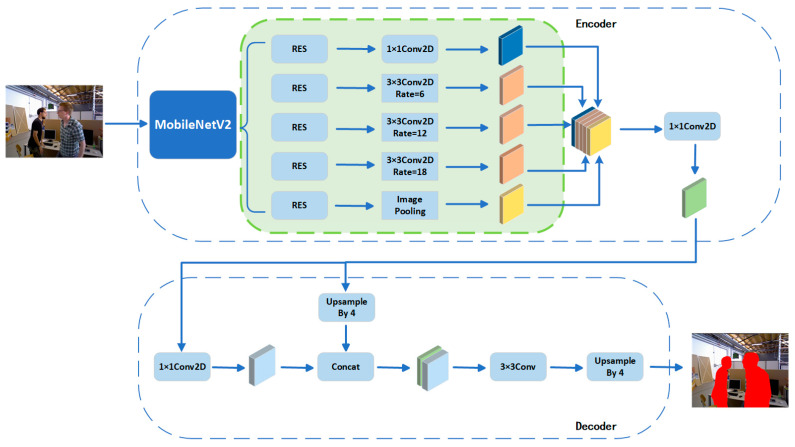
The schematic of the overall structure of Deeplabv3pro.

Conventional convolutional neural networks, especially in visual slam semantic segmentation tasks, may encounter problems such as limited receptive fields, inaccurate boundary localization, an insufficient ability to handle large-scale contextual information, and high training complexity. These limitations make it difficult for the network to capture a larger range of contextual information and fine-grained object details, leading to discontinuities and blurred boundaries in the segmentation results. Deeplabv3pro is based on the improved Deeplab [34,35] semantic segmentation model and is designed to address these issues; the overall structure is shown in Figure 2.

Deeplabv3pro combines a spatial pyramid pooling module with an encoder-decoder structure. Among them, the encoder module is the cornerstone of the whole structure, which is responsible for extracting deep semantic features from images. The main body of the encoder is a Deep Convolutional Neural Network (DCNN) [36,37], which can employ a variety of backbone networks such as ResNet [38], Xception [39], or MobileNetV2 [40], which provide the basic feature extraction capabilities for the model. In order to capture a wider range of contextual information without losing spatial resolution, the encoder uses inflated convolution instead of traditional convolution. Then, to further capture contextual information at multiple scales, the encoder deploys the Atrous Spatial Pyramid Pooling (ASPP) [41] module after the DCNN.ASPP achieves the capture of a wide range of spatial information from fine to coarse using the inflated convolution at different sampling rates.

MobileNetV2 is a lightweight deep learning network proposed by the Google team in 2018, designed for mobile and embedded devices. The core idea is to use deeply separable convolution instead of traditional convolutional operations, thereby drastically reducing the number of parameters and computation of the model without significant loss of accuracy.

#### 3.2.2. Improvement of ASPP

**Figure 3:**
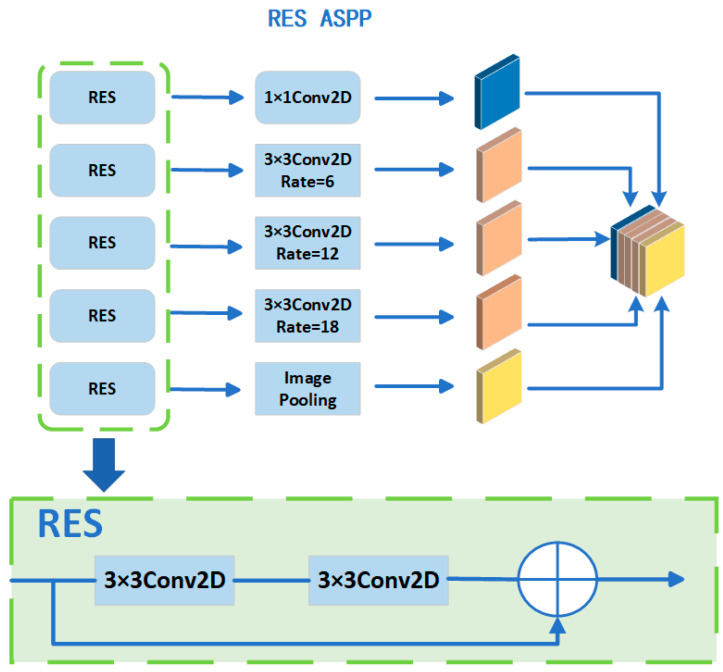
Modified ASPP framework. The upper part is the improved structure of the ASPP module, and the lower part is the newly added ResidualBlock.

In the encoder module of a deep learning framework, the Atrous Spatial Pyramid Pooling (ASPP) module is integral for multi-scale contextual information capture, a critical aspect for tasks such as image segmentation. ASPP functions through employing atrous convolutions at varied rates, enabling effective processing of features across multiple scales. The performance of ASPP, in terms of efficiency and accuracy, markedly influences the network’s ability to analyze and segment images with complex features and diverse scales. However, ASPP confronts challenges like gradient vanishing in the initial training phase of deep neural architectures, significantly impeding the learning of intricate or sophisticated features. In response, we improved the ASPP module by introducing ResidualBlock, which provides a simpler and more direct path to forward and backward propagate information through its residual connections, which helps prevent the gradient vanishing problem in deep networks. Second, this structure allows the model to learn incremental or “residual” representations of features, which is often easier than learning absolute representations directly. Finally, by adding such a structure to each inflated convolution, we can enhance the feature extraction capability of the model at various spatial scales, thus making it more robust and expressive, especially when dealing with image content with different scales and contexts. The improved result is shown in Figure 3.

ResidualBlock first defines two 3 × 3 convolutional layers, each following a batch normalization layer. During forward propagation, it first saves the inputs as residuals (residuals). The input is first passed through the first convolutional layer, the batch normalization layer, and then the ReLU activation function is applied. Next, the result passes through the second convolutional layer and another batch normalization layer. After this, the original residuals (i.e., the input) are added back to the output. Finally, the ReLU activation function is applied again, and the result is returned

When the feature maps generated by MobileNetV2 are sent to ASPP for processing, compared to the original ASPP, a ResidualBlock is added before each convolution to optimize feature extraction and avoid the gradient vanishing problem. Then, 1 × 1 convolutions with expansions of 6, 12, and 18 are performed, and a global average pooling operation is performed. The five feature maps are finally spliced in the channel dimension and feature fusion is achieved by 1 × 1 convolution, thus ensuring that features at different scales are effectively integrated to obtain a feature map containing high-level semantic features.

### 3.3. Multi-View Geometry Segmentation Module

Most dynamic objects can be segmented by Deeplabv3pro because they are trained to be a priori dynamic targets. However, there are still some limitations in some scenarios; some targets that are designated as static objects are often difficult to be detected when they move along with the a priori objects, for example, a person walks with a book in his hand or sits on a chair and moves around, then the book or the chair should be redefined as a dynamic target and rejected, and if it is not detected in time, then the accuracy of the whole system will receive a relatively large interference.

**Figure 4:**
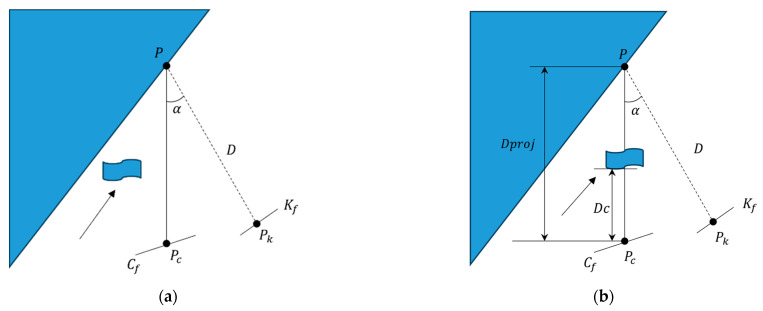
Multi-view geometry. (**a**) represents a keypoint identified as a static point, and (**b**) represents a keypoint identified as a dynamic point.

In order to solve this problem, this study proposed a dynamic feature point detection algorithm based on a multi-view geometry approach. As shown in Figure 4. Firstly, by projecting the map point cloud to the current frame, we can use the difference in viewpoints as well as the change in depth values to distinguish between dynamic and static targets. Specifically, for the current input frame $C_{f}$, we selected the keyframe $K_{f}$ that has a high degree of overlap with it, and projected the keypoints $P_{k}$ of the keyframe to the current frame $C_{f}$, so as to obtain the projection points $P_{c}$ and their corresponding projection depths Dproj. Next, we calculated the parallax angle α that was formed between n each keypoint $P_{k}$ and its projection point $P_{c}$. In the TUM dataset, it was observed that due to differences in viewpoints, static objects were often misclassified as dynamic when α exceeded 30 degrees. Therefore, we set the threshold of α at 30 degrees [12,42], considering points exceeding this threshold as dynamic. In addition, it was necessary to calculate the depth difference ΔD=Dproj−Dc, where Dc denotes the depth of the key point in the current frame. When ΔD was close to 0, the point could be identified as static; while when ΔD was significantly greater than 0, we considered the point to be dynamic.

This approach is highly advantageous for SLAM systems as it enables a more effective differentiation between static and dynamic objects. By precisely determining the dynamic nature of objects, this approach significantly enhances the accuracy and reliability of the map construction and localization process. It helps to reduce localization errors caused by misclassified objects, such as misclassifying a dynamic object as a static one. Additionally, it enhances the system’s adaptability in complex and changing real world environments. This method of dynamic object segmentation based on multi-view geometry is significant in enhancing the practicality and efficiency of SLAM.

## 4. Experimental and Analysis

### 4.1. Experimental Setup

This section conducts a number of experiments to evaluate the effectiveness of the ADM-SLAM system in a dynamic environment. In these experiments, the experimental environment used was Ubuntu 20.04, GeForce RTX 3070 graphics card, 8 GB video memory, 20-core 12th Gen Intel(R) Core(TM) i7-12700H CPU, and 16 GB RAM.

Experiment 1: We used Mur-Artal’s algorithm (quadtree algorithm) used in ORB-SLAM and the SSC algorithm in this paper for comparison experiments in the EuroC dataset [43] and the Tum RGB-D dataset.

**Figure 5:**
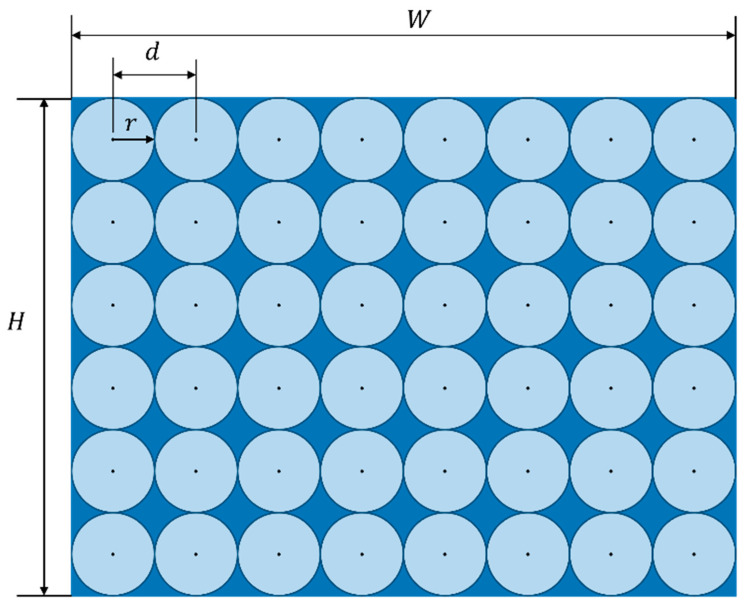
The schematic of the evaluation of homogeneity distribution coefficients.

To evaluate the impact of the SSC algorithm on improving local feature point homogenization, this paper used the homogeneity evaluation method proposed by Song et al. [44]. The method calculated homogeneity distribution coefficients based on the pixel distances between feature points and their nearest neighbors. The principle of this evaluation method is shown in Figure 5.

To distribute n feature points uniformly on a picture, the best scheme is to spread n circles with radius *r* all over the picture without overlapping. The position of the center of each circle was equivalent to the position of each feature point, as shown in the Figure 5. The distance between each feature point and its nearest neighbor is always d = 2*r*. The distance *d* was calculated as follows:

(13) $$d=H∕\sqrt{\frac{H\cdot n}{W}}=W∕\sqrt{\frac{W\cdot n}{H}}$$

In the above equation, H represents the pixel height of the image, W represents the pixel width of the image, and n represents the number of target feature points.

The most important feature of the template is that the distance between the feature point and the nearest feature point is d. From this, we can obtain the method of calculating the uniformity coefficient of the distribution of the feature points: firstly, we find out the distance $d_{i}$ between each feature point and its neighbouring feature points, and then we compare it with the template distance d, and then finally we find out the average value, the uniformity coefficient is:

(14) $$C=\frac{1}{n}\overset{n}{\underset{i=1}{\sum }}\frac{\left|d_{i}-d\right|}{d}$$

In the above equation, C represents the uniformity evaluation coefficient; $d_{i}$ represents the distance between the ith feature point and its neighboring feature points. A smaller value of the uniformity coefficient C indicates a better uniformity effect.

However, it is difficult to obtain high-quality images during SLAM, so it is necessary to test the feature point homogenization algorithm for different quality images to determine whether the feature point homogenization algorithm meets the requirements.

Experiment 2: In this experiment, we used Deeplabv3pro with the original deeplabv3 to validate its semantic segmentation performance in the Tum RGB-D dataset and to test its segmentation effect on dynamic objects.

In this experiment, we used the Pascal VOC2012 dataset [45] to test the improved image semantic segmentation model with the purpose of evaluating its real-time performance and accuracy. As evaluation indicators, we chose the segmentation speed and average intersection-over-union ratio (mIoU). mIoU is an important indicator used to measure the segmentation accuracy of the model. It is calculated by calculating the ratio of the intersection and union of the predicted value and the true value set. The calculation method of mIoU is as shown in the formula, where n represents the number of categories, TP is the number of correct predictions, FN is the number of missed detections, and FP is the number of false detections.

(15) $$mIoU=\frac{1}{n+1}\overset{n}{\underset{i=0}{\sum }}\frac{TP}{FN+FP-TP}$$

The basis of this model training platform was the Ubuntu 20.04 operating system. In terms of hardware configuration, the processor we used was the 12th Gen Intel(R) Core(TM) i7-12700H, equipped with NVIDIA GeForce GTX 3070 graphics card. In terms of software configuration, model training was based on the TensorFlow deep learning framework. Key training parameter settings included the following: the batch size was set to 8, the initial learning rate was set to 0.001, and the weight decay rate was set to 0.0001. These parameters together ensure the efficiency and effectiveness of model training.

Experiment 3: In this experiment, we conducted tests on the public TUM RGB-D dataset using our ADM-SLAM system, comparing it with ORB-SLAM2. The evaluation metrics included the Absolute Pose Error (ATE) and the Relative Pose Error (RPE). This experiment aimed to assess the performance of ADM-SLAM in dynamic environments.

Experiment 4: This experiment also utilized the public TUM RGB-D dataset, where we compared ADM-SLAM with Dyna-SLAM and DS-SLAM. The evaluation metrics were the Absolute Trajectory Error (ATE) and real-time performance. The purpose of this experiment was to further explore the effectiveness of ADM-SLAM in dynamic scenes and its feasibility in real-time applications.

### 4.2. Experiment 1: Evaluation of Adaptive Feature Point Homogenisation Extraction Performance 

Firstly, we used Mur-Artal’s algorithm, as employed in ORB-SLAM, along with the adaptive feature point homogenization extraction algorithm proposed in this paper, to analyze the EuroC dataset and the Tum RGB-D dataset of 7 video sequences. Specifically, we extracted 50, 250, and 500 key points from EuroC Machine Hall (01, 02, 03) and Tum RGB D frl (xyz, rpm, 360, desk), respectively. We then tested and averaged the results of 30 experiments to calculate the Uniformity coefficient for each frame. The specific comparison results are shown in Table 1, which provides a uniformity comparison of the quadtree and SSC algorithm.

**Figure 6:**
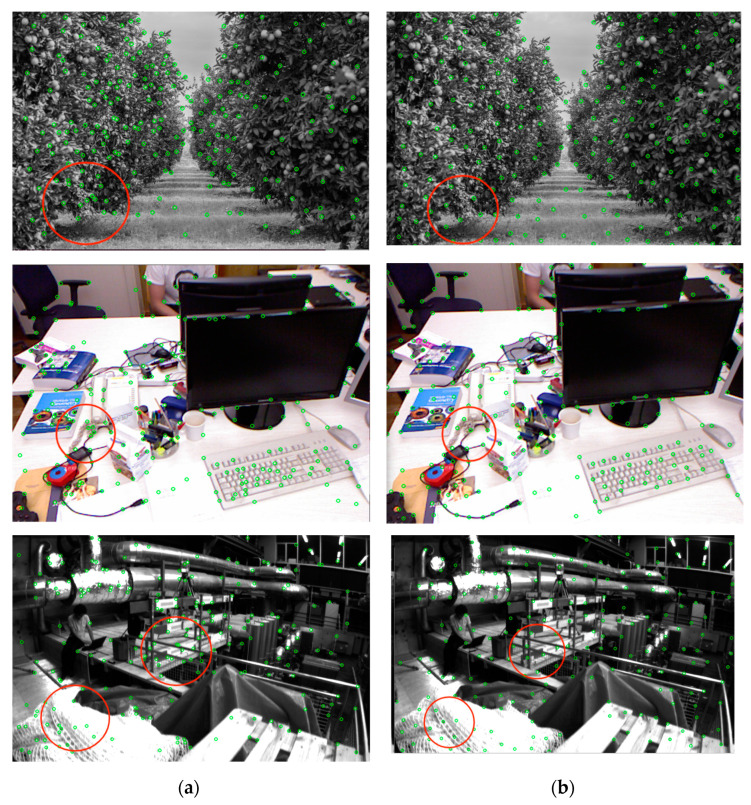
The quadtree and the SSC are compared on different data sets. The difference is more obvious in the places selected by the red circle. (**a**) Quadtree; (**b**) SSC.

Meanwhile, this paper compares the feature points extracted by quadtree algorithm and SSC (suppression via square coverage) algorithm in Tum RGB-D dataset open-source image data respectively with 250 feature points and the results of feature extraction time are shown in Table 2. The results of both algorithms for the same image to extract ORB feature points are shown below Figure 6.

Table 1 compares the performance of the quadtree algorithm in ORB-SLAM and our proposed SSC algorithm in terms of uniformity. The results clearly show that our algorithm significantly outperformed the quadtree algorithm in ORB-SLAM in terms of uniformity. The uniformity of the SSC algorithm was better when the number of extracted target points was smaller, and the effect embodied in this paper’s algorithm was not obvious when the number of extracted target points was higher. The current visual SLAM based on the feature point method extracted about 1000 feature points per frame, and after image pyramid layering, the number of feature points in the bottom layer was about 250. The average uniformity coefficient per frame and the time consumed for feature extraction per frame for a target feature point number of 250 were significantly improved, which provides more spatial feature information and saves computation time for the visual SLAM; this also helps to improve the operational performance of SLAM.

As shown in Table 2, our SSC algorithm had a shorter extraction time and was more efficient than the quadtree algorithm in ORB-SLAM.

In Figure 6, as marked by the red circles, we compared the feature points extracted from the same local information using different methods. The results demonstrate that while the quadtree normalization method can distribute feature points uniformly across the entire image, it leads to excessive density and redundancy in local areas, failing to effectively represent the spatial information of the image. In contrast, our method not only achieves a uniform distribution of feature points across the entire image but also more effectively highlights the key features of the image.

### 4.3. Experiment 2: Evaluation of Deeplabv3pro Semantic Segmentation Performance

**Figure 7:**
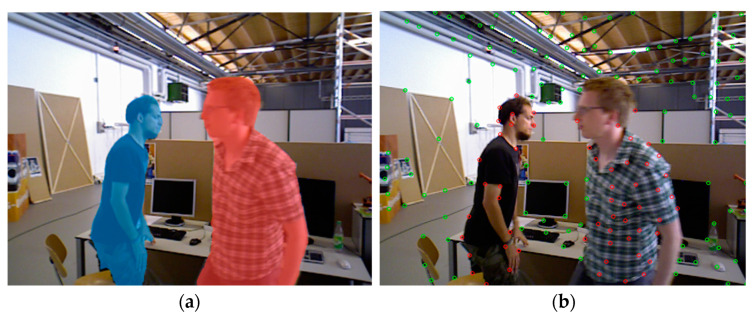
Segment images and extract feature points from dynamic scenes. (**a**) is the result after improved Deeplabv3pro segmentation, masks of different colors represent segmented objects; and (**b**) is the result of using SSC to extract feature points from the segmented image. Red represents dynamic feature points, green points represent static feature points.

The original ORB-SLAM2 has relatively weak processing capabilities in the face of dynamic environments. The RANSC of the algorithm itself can only remove a small number of feature points on dynamic objects and cannot handle high-intensity dynamic environments. To improve the robustness of the algorithm in dynamic environments, this article adds a semantic segmentation module based on the original ORB-SLAM2 algorithm and applies the semantic segmentation module in the dynamic point elimination process. The algorithm in this article uses the Deeplabv3pro semantic segmentation network to segment each frame of an RGB image and obtain the mask of the corresponding area. This article sets people as dynamic prior objects, so the feature points of people are the first to eliminate. The dynamic point rendering is shown in Figure 7. Semantic segmentation was used to remove dynamic feature points. Figure 7a is the semantic mask image obtained after segmentation. Figure 7b is the image with dynamic feature points removed. Among them, the dynamic feature points of red points. The green points are static feature points.

**Figure 8:**
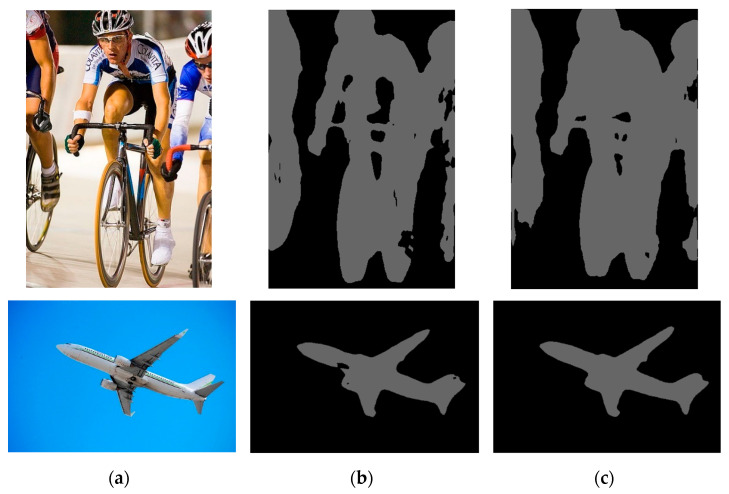
Images segmented using different models; (**a**) is the original input image, (**b**) is the image segmented by deeplab v3+, (**c**) is the image segmented by improved deeplab v3+.

As shown in Table 3 and Figure 8, the experimental results show that the Deeplabv3pro model showed significant performance improvement when processing images with multiple targets or clear outlines. Compared with the original model, it as more accurate in capturing and segmenting rough contours, especially the sensitivity and detail recognition capabilities of the edge parts of the image have been significantly improved. The key improvement lies in the backbone network. Using the more lightweight MobileNetV2 and optimizing the residual operation of ASPP, the processing efficiency of the model has been significantly improved. These improvements resulted in the model’s memory footprint being reduced by approximately 94.62%, while the processing time for a single image was also reduced by approximately 86.80%. This not only means higher operating efficiency, but also shows that the overall performance of the model has been significantly improved, meeting the strict real-time requirements of the image segmentation task and demonstrating the advantages of the improved model in resource utilization and speed.

### 4.4. Experiment 3: Evaluation of ADM-SLAM and ORB-SLAM2 Performance in Dynamic Environment

In this study, we aimed to evaluate the ADM-SLAM system’s ability to handle dynamic environments and adopted the TUM RGB-D dataset commonly used in the slam field, created by the computer vision team of the Technical University of Munich in Germany, covering 39 indoor scenes. Image sets and corresponding precise camera trajectories. This dataset provides a wide range of evaluation scenarios for visual SLAM performance, including “sitting” low-dynamic sequences and “walking” and other eight high-dynamic sequences. As an advanced extension of ORB SLAM2, the ADM-SLAM system first performs a performance comparison with ORB-SLAM2. We used the evo toolkit to compare the absolute pose error (ATE) and relative pose error (RPE) of the ORB-SLAM2 and ADM-SLAM algorithms to quantitatively analyze the positioning accuracy and overall system performance. ATE measures the global consistency of the trajectory, while RPE focuses on measuring its own translational and rotational drift. Furthermore, we use the root mean square error (RMSE), mean, and the standard deviation error (Std) to evaluate the accuracy, robustness, and stability of the system, respectively.

According to the data in Table 4 and Table 5, compared with ORB-SLAM2, the performance of the ADM-SLAM algorithm in this study has been significantly improved in dynamic scenes. Especially in highly dynamic scenes, ATE can be reduced by up to 97%. In low-dynamic scenes, due to the optimized design of ORB-SLAM2 itself for low-dynamic environments, the improvement effect of ADM-SLAM relative to high-dynamic scenes has been weakened, but it still shows a robust performance improvement.

**Figure 9:**
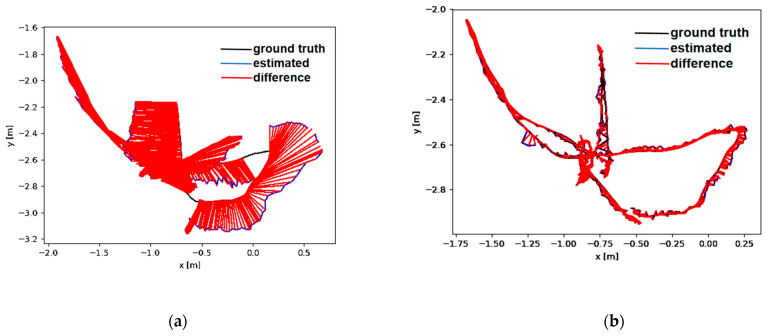

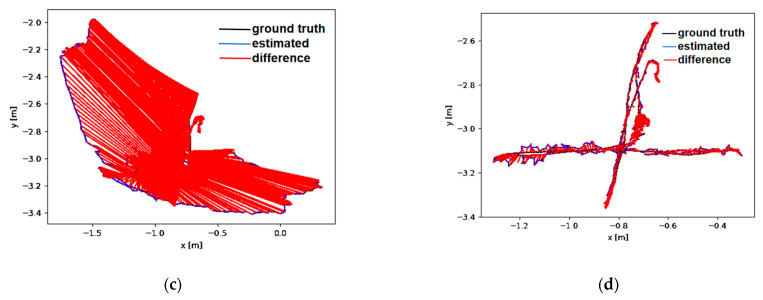
Trajectories of highly dynamic sequences. The black line is the ground truth, the blue line is the estimated value, and the red line is the difference. (**a**,**c**) is the result of ORB-SLAM2, (**b**,**d**) is the result of ADM-SLAM. (**a**,**b**) fr3_w_xyz; (**c**,**d**) fr3_w_half.

To accurately visualize and demonstrate the significant advantages of our algorithm over ORB-SLAM2 in processing high-dynamic scenes, we plotted the Absolute Trajectory Error (ATE) graphs for high-dynamic sequences, as shown in Figure 9. In these images, the upper half depicts the performance of the original ORB-SLAM2 algorithm, while the lower half displays the performance of our study’s algorithm. In the graphs, the black curve represents the actual camera trajectory, the blue curve shows the estimated trajectory, and the red segments indicate the error between the two. The length of the red segments is inversely proportional to the algorithm’s accuracy; shorter segments denote higher accuracy.

**Figure 10:**
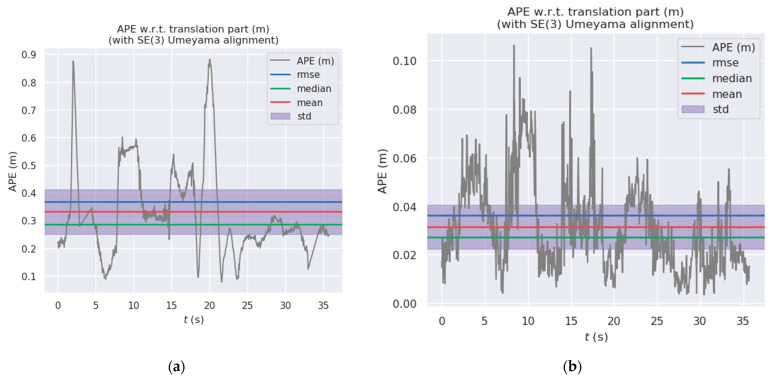
Absolute trajectory error distribution plot of fr3_w_half sequence. (**a**) APE of ORB-SLAM2; (**b**) APE of ADM-SLAM.

**Figure 11:**
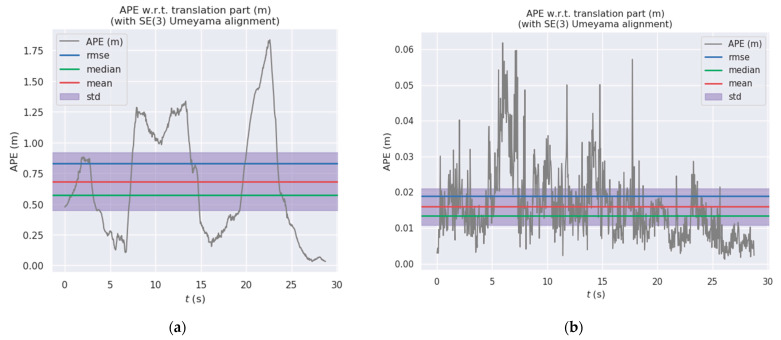
Absolute trajectory error distribution plots of fr3_w_half sequence. (**a**) APE of ORB-SLAM2; (**b**) APE of ADM-SLAM.

Figure 10 and Figure 11 show the distribution diagrams of the absolute trajectory errors of ADM-SLAM and ORB-SLAM2 for the walking_half and walking_xyz sequences, respectively, where Figure 10a and Figure 11a are the ATE of ORB-SLAM2, and Figure 10b and Figure 11b are the ATE of ADM-SLAM.

**Figure 12:**
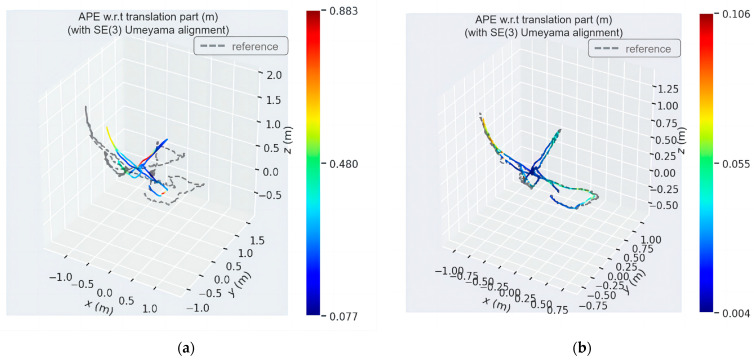
Three-dimensional space absolute trajectory error distribution diagram of fr3_w_half sequence. (**a**) APE of ORB-SLAM2; (**b**) APE of ADM-SLAM.

**Figure 13:**
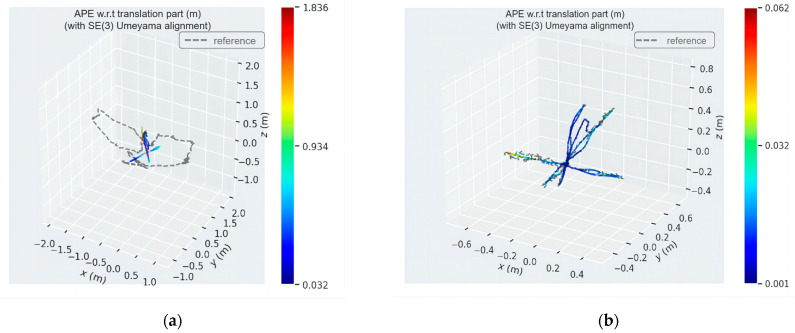
Three-dimensional space absolute trajectory error distribution plots of fr3_w_half sequence. (**a**) APE of ORB-SLAM2; (**b**) APE of ADM-SLAM.

Figure 12 and Figure 13 show the absolute trajectory error plots of ADM-SLAM and ORB-SLAM2 in three-dimensional space for the walking_half and walking_xyz sequences, respectively, where Figure 12a and Figure 13a are the APE of ORB-SLAM2, and Figure 12b and Figure 13b are the APE of ADM-SLAM.

The analysis of high-dynamic scenes reveals that the ORB-SLAM2 algorithm exhibited significant errors in camera trajectory estimation, evident in the notable trajectory deviation. In contrast, our study’s algorithm significantly reduced the error in camera motion trajectory, demonstrating smaller trajectory deviations. This comparative result effectively validates the superior performance of our method in high-dynamic environments, highlighting its significant advantages in accurately tracking camera trajectories.

Although the improvement of the algorithm focuses on high-dynamic environment sequences, we still conducted tests under low-dynamic environment sequences. The test results show that in low-dynamic environment sequences, ADM-SLAM had a lower mean square of ATE than the ORB-SLAM2 algorithm in low-dynamic environment sequences. The root error was reduced by 28.61% on average, and the standard deviation was reduced by 21.96%. The results prove that the algorithm also has good stability in low-dynamic environments.

### 4.5. Experiment 4: Evaluate the Performance of ADM-SLAM and Other SLAM Dynamic Environment Algorithms

To further explore the performance of ADM-SLAM in dynamic environments, this study conducted a series of comparative experiments involving DS-SLAM and DynaSLAM. The core evaluation metrics of these experiments include the Root Mean Square Error (RMSE) and the standard deviation (std) of the Absolute Trajectory Error (ATE). The experimental data and detailed analysis results are presented in Table 6. The results show that in dynamic test sequences, the performance of ADM-SLAM was significantly superior to DS-SLAM and Dyna-SLAM, demonstrating its exceptional adaptability and accuracy in complex dynamic settings.

Real-time performance holds a crucial position in the practical application of SLAM systems, directly affecting the algorithm’s usability and superiority. To comprehensively assess the real-time performance of ADM-SLAM, we selected DS-SLAM, DynaSLAM, and ORB-SLAM2 for comparison, focusing on the time required to process each frame of different sequences. The related experimental data are detailed in Table 7. The results indicate that although ADM-SLAM introduces additional object detection and processing steps, resulting in slightly longer processing times than ORB-SLAM2, its real-time performance remains superior to other SLAM methods designed for dynamic scenes. This demonstrates its capability to effectively handle dynamic environments while maintaining a lower runtime.

## 5. Discussion and Conclusions

This study, through the development and implementation of ADM-SLAM, advances the field of SLAM systems in dynamic environments. By integrating a novel lightweight semantic segmentation with multi-view geometric segmentation techniques, ADM-SLAM effectively eliminates the adverse impacts of dynamic objects on SLAM systems’ positioning accuracy. This system marks a significant improvement over ORB-SLAM2 by enhancing dynamic feature point detection through the incorporation of semantic and multi-view geometric methods. Furthermore, the Adaptive Non-Maximum Suppression (ANMS) algorithm SSC is innovatively used to homogenize the distribution of feature points, resulting in reduced extraction time.

The experimental results on the TUM RGB-D dataset underscore ADM-SLAM’s superior performance in high-dynamic environments. Not only does it significantly outpace the original ORB-SLAM2 algorithm, but it also surpasses existing dynamic environment localization methods in both positioning accuracy and real-time operation. This accomplishment addresses a crucial scientific problem in the field of robotics and autonomous navigation systems, where dynamic environments pose a persistent challenge.

Despite these advances, areas for future enhancement remain. The processing speed of semantic segmentation and geometric threading of images are pivotal to the system’s overall real-time performance. Continued refinement and optimization of the semantic segmentation network will further improve the precision of dynamic object handling. Future research will aim to enhance the identification and processing of dynamic points, including the introduction of new threads for constructing richer semantic maps. These improvements are expected to not only boost the system’s performance and adaptability but also extend its applicability to more complex and variable real-world scenarios.

## Figures and Tables

**Table 1:** Uniformity comparison of quadtree and SSC algorithm.

Target Feature Points	Algorithm	MH01	MH02	MH03	fr1/xyz	fr1/rpy	fr1/360	fr1/Desk
50	Quadtree	0.5054	0.5031	0.4982	0.4764	0.4746	0.4894	0.4831
	SSC	0.2168	0.2250	0.2340	0.3016	0.3598	0.3455	0.3295
250	Quadtree	0.5159	0.5175	0.5185	0.5465	0.5637	0.5940	0.5558
	SSC	0.3278	0.3408	0.3788	0.4755	0.5579	0.5233	0.5102
500	Quadtree	0.5045	0.5077	0.5267	0.6103	0.6438	0.6826	0.6337
	SSC	0.4221	0.4383	0.4881	0.5860	0.6794	0.6634	0.6302

**Table 2:** Time-consuming comparison of quadtree and SSC algorithm (ms).

	MH01	MH02	MH03	XYZ	rpy	360	Desk
Quadtree	8.263	8.119	7.831	6.300	5.622	5.542	5.998
SSC	6.267	6.165	6.108	5.711	5.233	5.096	5.385
Improvement	24.2%	24.1%	22.0%	9.35%	6.91%	8.05%	10.22%

**Table 3:** Comparison of parameters before and after Deeplabv3pro improvement.

Model	MioU (%)	Time (ms)	Size (M)
Deeplab v3	70.06	189.4	439
Deeplab v3 pro	73.44	25	23.6
Improvement/%	4.82	86.80	94.62

**Table 4:** Comparison of ATE between ORB-SLAM2 and ADM-SLAM in tum sequences.

Sequence	ORB-SLAM2/m			ADM-SLAM/m			Improvement/%		
	Rmse	Mean	Std	Rmse	Mean	Std	Rmse	Mean	Std
walking_xyz	0.8293	0.6834	0.4698	0.0189	0.0159	0.0102	97.72	97.67	97.83
walking_half	0.3683	0.3307	0.1621	0.0363	0.0314	0.0182	90.14	90.50	88.77
walking_static	0.4314	0.3852	0.1943	0.0103	0.0094	0.0043	97.61	97.56	97.79
walking_rpy	0.6519	0.5719	0.3131	0.0298	0.0242	0.0174	95.43	95.76	94.44
sistting_xyz	0.0157	0.0097	0.0136	0.0107	0.0079	0.0046	31.85	18.56	66.17
sisting_half	0.0466	0.0179	0.0367	0.0200	0.0132	0.0091	57.08	26.25	75.21
sitting_static	0.0136	0.0122	0.0057	0.0060	0.0052	0.0030	55.88	57.37	47.37
sisting_rpy	0.0386	0.0166	0.0322	0.0242	0.0185	0.0156	37.30	6.02	35.54

**Table 5:** Comparison of RPE between ORB-SLAM2 and ADM-SLAM in tum sequences.

Sequence	ORBSLAM2/m			ADM-SLAM/m			Improvement/%		
	Rmse	Mean	Std	Rmse	Mean	Std	Rmse	Mean	Std
walking_xyz	0.0267	0.0218	0.0153	0.0132	0.0106	0.0077	50.56	51.38	49.67
walking_half	0.0323	0.0199	0.0254	0.0154	0.0123	0.0092	52.32	22.61	63.78
walking_static	0.0149	0.0101	0.0110	0.0066	0.0055	0.0037	55.70	45.54	66.36
walking_rpy	0.0278	0.0216	0.0175	0.0256	0.0201	0.0162	7.91	6.94	7.43
sistting_xyz	0.0126	0.0103	0.0058	0.0091	0.0080	0.0045	27.78	11.65	22.41
sisting_half	0.0188	0.0123	0.0191	0.0109	0.0093	0.0056	42.02	24.39	70.68
sitting_static	0.0145	0.0133	0.0057	0.0056	0.0049	0.0027	61.37	63.16	52.63
sisting_rpy	0.0202	0.0191	0.0131	0.0142	0.0109	0.0092	29.70	42.93	29.77

**Table 6:** Comparison results of ATE of different SLAM algorithms under highly dynamic sequences; bold indicates the best results.

Sequence	DS-SLAM		Dyna-SLAM		ADM-SLAM	
	RMSE	Std	RMSE	Std	RMSE	Std
walking_xyz	0.0262	0.0165	0.0236	0.0117	**0.0189**	**0.0102**
walking_half	0.0419	0.0262	0.0376	0.0232	**0.0363**	**0.0182**
walking_static	0.0192	0.0066	0.0193	0.0078	**0.0103**	**0.0043**
walking_rpy	0.4628	0.2306	0.2935	0.2314	**0.0298**	**0.0174**
sistting_xyz	0.0181	0.0132	0.0203	0.0087	**0.0107**	**0.0046**
sisting_half	0.0152	0.0059	**0.0126**	**0.0043**	0.0200	0.0091
sitting_static	**0.0056**	**0.0028**	0.0081	0.0041	0.0060	0.0030
sitting_rpy	**0.0236**	**0.0143**	0.0386	0.0215	0.0242	0.0156

**Table 7:** Comparison of real-time performance between ADM-SLAM and other dynamic scene SLAM methods.

Dataset	DS-SLAM	DynaSLAM	ADM-SLAM	ORB-SLAM2
walking_xyz	0.856	1.212	0.094	0.0384
walking_half	0.764	1.138	0.125	0.0402
walking_static	0.826	1.342	0.102	0.0390

## Data Availability

The research data used in this paper are from https://projects.asl.ethz.ch/datasets/doku.php?id=kmavvisualinertialdatasets (accessed on 12 February 2024) and https://vision.in.tum.de/data/datasets/rgbd-dataset/download (accessed on 12 February 2024).
